# Dietary inclusion of anthocyanin-rich black cane silage treated with ferrous sulfate heptahydrate reduces oxidative stress and promotes tender meat production in goats

**DOI:** 10.3389/fvets.2022.969321

**Published:** 2022-08-04

**Authors:** Rayudika Aprilia Patindra Purba, Ngo Thi Minh Suong, Siwaporn Paengkoum, Jan Thomas Schonewille, Pramote Paengkoum

**Affiliations:** ^1^School of Animal Technology and Innovation, Institute of Agricultural Technology, Suranaree University of Technology, Nakhon Ratchasima, Thailand; ^2^School of Animal Sciences, Agriculture Department, Can Tho University, Can Tho, Vietnam; ^3^Program in Agriculture, Faculty of Science and Technology, Nakhon Ratchasima Rajabhat University, Nakhon Ratchasima, Thailand; ^4^Department of Population Health Sciences, Faculty of Veterinary Medicine, Utrecht University, Utrecht, Netherlands

**Keywords:** anthocyanin, antioxidant capacity, agricultural waste, carcass characteristics, fermentation, microbial rumen, iron-treated silage

## Abstract

Pre-treating anthocyanin-rich black cane with ferrous sulfate heptahydrate (FSH) produces high-quality silage with anthocyanin and nutritional losses. However, it's unclear how to apply this to studies on how FSH-treated silage affects animal performance and meat quality. Therefore, this study aimed to investigate the effects of a standard total mixed ration (TMR) containing anthocyanin-rich black cane silages either with or without dilutions of FSH on animal performance, blood biochemical indices, rumen fermentation, microbial community, and carcass characteristics in meat goats. Forty healthy crossbred Thai-native Anglo-Nubian male goats (14.42 ± 1.4 kg) were used to compare the feasibility of using anthocyanin-rich black cane silage (ABS) as a functional feed resource as opposed to anthocyanin-rich black cane treated with 0.030% commercial FSH silage (ABSF). All goats received a 90-day routine feeding of two isocaloric and isonitrogenous experimental diets: the control group received TMR containing 50% anthocyanin-rich black cane silage (ABS; *n* = 20), and one group received TMR containing 50% FSH-treating anthocyanin-rich black cane (ABSF; *n* = 20). As performance indicators, average daily weight gain (ADG) and dry matter intake (DMI) were measured. Samples of meat, blood, and rumen were taken at the end of the experiment. There were no differences in final body weight, ADG, DMI, or ADG/DMI between the two groups. The ABSF group did not differ from the ABS group in terms of rumen pH, but the ABSF had a tendency to lower rumen ammonia N levels, and to higher total volatile fatty acid (VFA) concentrations. Individual VFA concentrations differed, with the ABSF group having more *Ruminococcus albus* and the ABS group having more methanogenic bacteria. Blood biochemical indices differed, with the ABSF group having lower TBARS concentrations and the ABS group having lower TAC, SOD, CAT, GSH-Px, and GSH-Rx concentrations. In comparison to goat meat from the ABS group, goat meat from the ABSF group contained more intramuscular fat and was more tender. The current results indicate that the feeding of a TMR containing 50% anthocyanin-rich black cane, along with FSH pre-treatment prior to ensiling, reduces oxidative stress and promotes the production of tender meat without affecting animal performance.

## Introduction

Ensiling has been proposed as an efficient method of preserving roughage in light of the fact that such agricultural activities pose a significant disposal problem, e.g., decomposition or open burning. However, it necessitates a suitable effort prior to ensiling because the breakdown of biomass during anaerobic fermentation results in functional microorganisms and chemicals that reduce nutrient loss in addition to harmful substances (1). Using iron dilutions can help significantly with forage preservation (2). These additives can contribute to the retention of nutrients in silage, hence strengthening its feed value and improving the production efficiency of ruminants (3, 4). Several direct-fed iron sources had been shown to improve the health and productivity of small ruminants (3, 5). Direct-fed irons are typically given orally as an encapsulated capsule or incorporated into the diet (6). Of our knowledge, there have been few studies on the viability of direct-fed iron, especially ferrous sulfate heptahydrate (FSH), for goats; hence, introducing direct fed FSH into the diet *via* silage could be a novel strategy to administering these additives to goats.

In recent years, oxidative stress generated by heat stress has become a well-known limiting factor for animal performance and health in tropical regions (7, 8). From a practical standpoint, the feeding of anthocyanin-rich black cane (*Saccharum sinensis* Robx.) can be regarded as a beneficial feed resource in tropical ruminant nutrition, including beneficial anthocyanins for meat goats (9, 10). Anthocyanin-rich black cane is a by-product of agricultural production, and the total yield of anthocyanin-rich black cane is estimated to be 1.2 × 10^7^ metric tons of dry matter (DM) in 2021 from 1.58 × 10^6^ ha in Thailand (9, 11). However, the highly lignified components of anthocyanin-rich black cane reduce the bioaccessibility of anthocyanin during rumen feeding. In addition, earlier studies demonstrated that the incorporation of anthocyanin-rich black cane with dilutions of FSH at levels ranging from 0.015 to 0.030% on fresh weight (FW) into anthocyanin-rich black cane silages improved the nutritional balance with massively decreased lignin contents, as well as modulated anthocyanin stability during ensiling, and *in vitro* ruminal fermentation of anthocyanin-rich black cane silages (1, 2). These findings support the notion that using those FSH levels might aid the current study in terms of decreasing the lignin content of anthocyanin-rich black cane silages prior to feeding. FSH is a beneficial inorganic salt that acts as a catalyst in enzymatic hydrolysis and fermentation during ensiling (1, 2). Besides, anthocyanin in black cane-treated FSH was discovered to be stable throughout rumen fermentation (2). This such benefit of the black cane's anthocyanin is resistant to ruminal digestion and, as a result, may be absorbed by small intestine. As a consequence of this, anthocyanins are made available for absorption and may be able to utilize their antioxidative properties to provide protection against the damaging effects of oxidative stress. Furthermore, ensiling anthocyanin-rich black cane with 0.030% FSH included increased the acetate to propionate ratio, as well as a shift in the structure and relative abundance of the microbial population in incubated rumen fluids *in vitro* (1, 2). Similarly, recent studies showed that the microbial community in slaughtered goat rumen fluid was shifted to have a tendency to increase the acetate to propionate ratio, as influencing anthocyanin fractions from purple corn (12) and black cane (9). Increases in ruminal acetic acid production caused by anthocyanins may make more substrate available for *de-novo* fat synthesis (13). However, the molecular weight of anthocyanins can differ between plant species or during processing (14, 15), which makes it difficult to directly extrapolate the findings reported by Tian et al. (12) or Suong et al. (9).

**Figure 1:**
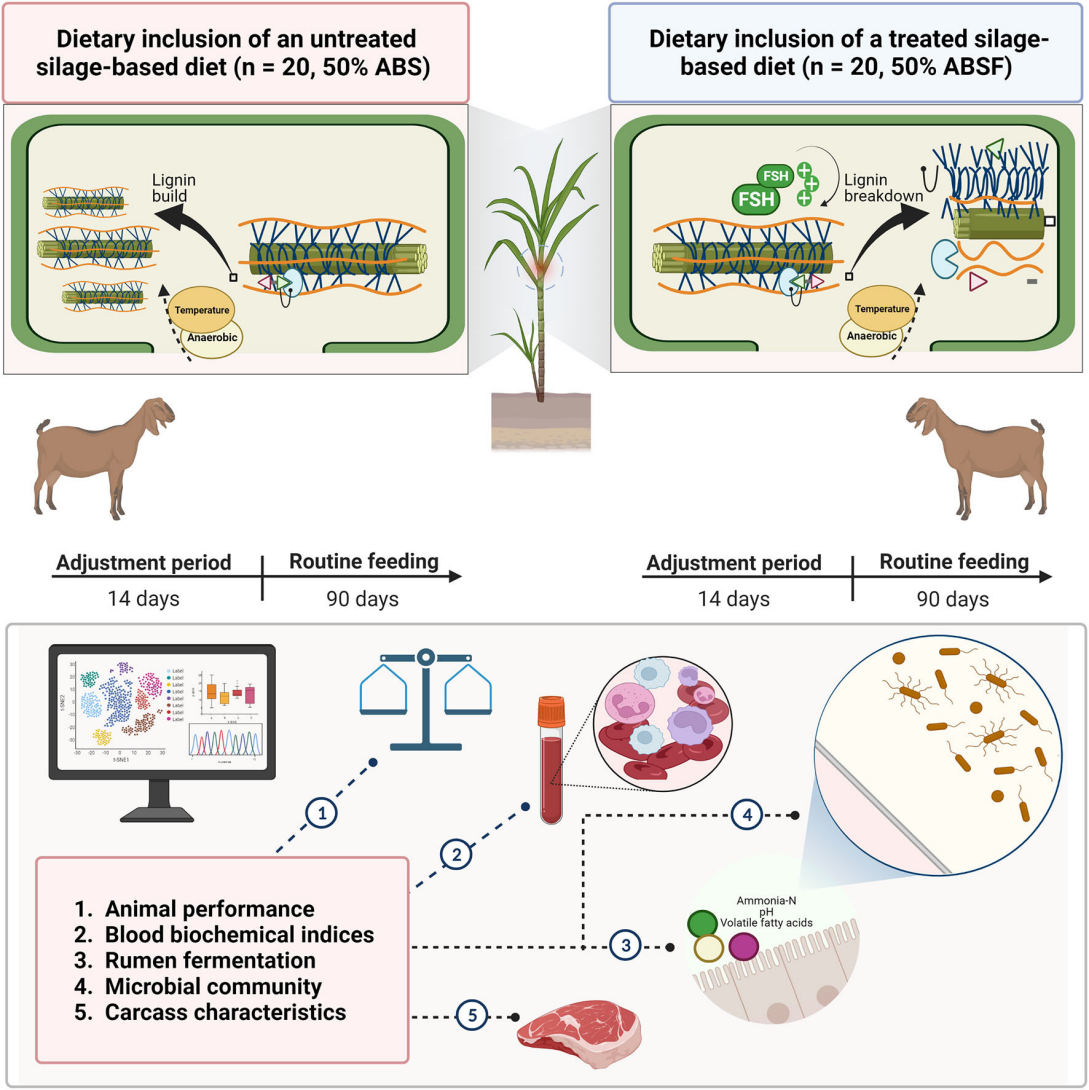
A 90-day routine feeding was conducted to investigate the feasibility of using anthocyanin-rich black cane silage (ABS) as a functional feed resource vs. anthocyanin-rich black cane with 0.03% FSH (ABSF) in healthy crossbred Thai-native Anglo-Nubian male goats.

To date, however, evaluation of anthocyanin-rich black cane silage treated with FSH has been limited to laboratory scale silos and *in vitro* laboratory procedures (1, 2). It is unclear how to apply current *in vitro* results to studies on how FSH-treated silage affects animal performance and meat quality in practice. Therefore, the objective of present study was to investigate the effects of a standard total mixed ration (TMR) containing anthocyanin-rich black cane silages either with or without dilutions of FSH on animal performance, blood biochemical indices, rumen fermentation, microbial community, and carcass characteristics in meat goats (Figure 1).

## Materials and methods

### Roughage harvesting and ensiling

Anthocyanin-rich black cane (cane hybrid: *Saccharum spontaneum* x *Saccharum officinarum*) was cultivated and harvested at the Suranaree University of Technology (SUT) goat and sheep research farm in Nakhon Ratchasima, Thailand (14°52'49.1“N, 102°00'4.9”E, 243 m above sea level). An experimental field study was carried out between August 2017 and January 2018, during the monsoon season, with plant management in accordance with Suong (16). Fresh anthocyanin-rich black cane was sampled on the 60th d after 120 d of regrowth by cutting well above the soil surface (10 cm above ground level). Six random spots in the field were sampled for the fresh materials. The gathered materials were then chopped to a length of 2–3 cm using a crop cutter and homogenized thoroughly. Following two treatments, anthocyanin-rich black cane materials were ensiled without wilting: no additives or anthocyanin-rich black cane only (ABS) and anthocyanin-rich black cane treated with 0.030% commercial ferrous sulfate heptahydrate (FeSO_4_xH_2_O; Merck KGaA, Darmstadt, Germany) on fresh weight (ABSF). The dosage level was selected based on previous studies and animal safety assessments (2, 17). In brief, 0.030 g FSH was prepared in a Beaker glass and stirrer by adding up to 1000 mL of water. The prepared FSH mixture was then sprayed onto a portion of the ABSF (1 kg) and mixed until homogeneity was achieved. The prepared silages were stored at 25–27°C in plastic drums (Misumi, Bangkok, Thailand). These procedures were similar to the ABS portion but did not include the administration of FSH. Both silages fermented well after 90 d of ensiling. This was shown by a sensory evaluation and a pH value measured with a portable pH meter (Oakton pH 700, Long Branch, New Jersey, NJ, USA) when the drum containing the silage was opened. The composition and fermentation characteristics of ABS and ABSF are detailed in Supplementary Table 1.

### Goats and experimental diets

Forty healthy crossbred Thai-native Anglo-Nubian male goats with a body weight (BW) of 14.42 kg (±1.4 SD) were selected from a total of 400 goats raised by cooperating Thai farmers. Individually housing and randomly assigning the goats to one of two experimental diets (Table 1) were: 1) Untreated silage-based diet (*n* = 20, 50% ABS) or 2) treated silage-based diet (*n* = 20, 50% ABSF) using a completely randomized design. The remaining elements were identical to those used in an NRC-compliant basal total mixed ration (TMR) diet at 50:50 of roughage to concentrate ratio (R:C) level (18). Prior to the trial, the goats were acclimatized to routine feeding and their diets were properly adjusted during a 14-day adjustment period. The animals were fed experimental diets gradually during the adjustment phase. After 14 d, the experimental diets were fed to the animals on a daily basis for a total of 90 d. During the duration of the experiment, the average amount of feed consumed by the animals was ~ 95% of the total amount provided, which were ~ 465 g DM of TMR each day, divided into two equal amounts at 0700 and 1,600. The goats had unrestricted access to clean water and a block of trace mineral salt. To determine animal performance, feed refusals were recorded on a daily basis and utilized to determine DM intake (DMI). The BW of the goats was recorded in order to evaluate their growth performance throughout the routine feeding period.

**Table 1:** Ingredients and nutrient composition of experimental diets.

**Item**	**ABS**	**ABSF**
**Ingredient, DM basis**		
Anthocyanin-rich black cane silage	50.0	0.0
Anthocyanin-rich black cane silage treated with iron^a^	0.0	50.0
Cassava pulp	3.8	3.8
Cassava chip	19.5	19.5
Mineral mix^b^	0.7	0.7
Palm meal	14.0	14.0
Compound premix^c^	0.2	0.2
Rice bran	5.5	5.5
Soybean meal	4.0	4.0
Sulfur^d^	0.4	0.4
Sunflower oil	1.0	1.0
Urea	0.9	0.9
**Nutrient composition, DM basis**		
Metabolizable energy (Mcal/kg)	17.36	17.34
Crude protein (%)	11.61	11.64
Neutral detergent fiber (%)	52.93	52.87
Acid detergent fiber (%)	24.41	24.33
Neutral detergent lignin (%)	3.38	2.67
Hemicellulose (%)	28.52	28.54
Cellulose (%)	21.03	21.66
Ash (%)	10.53	11.05
Total anthocyanins (mg/g)	0.17	0.28
**Anthocyanin profile (% of total anthocyanins)**		
Cyanidin-3-glucoside	4.83	3.14
Pelargonidin-3-glucoside	7.24	5.62
Delphinidin	16.90	15.04
Peonidin-3-O-glucoside	16.21	14.71
Malvidin-3-O-glucoside	14.14	18.43
Cyanidin	25.17	16.86
Pelargonidin	1.72	1.49
Malvidin	13.79	24.71

a *Anthocyanin-rich black cane treated with 0.30% of ferrous sulfate heptahydrate*.

b *Contained (g/kg): NaCl (600), P (160), Ca (240)*.

c *Vitamin A (4,200.000 IU/kg), vitamin A3 (840,000 IU/kg), vitamin E (10,000 IU/kg), vitamin K3 (2 g/kg), vitamin B1 (2.4 g/kg), vitamin B2 (3.5 g/kg), vitamin B6 (1.8 g/kg), vitamin B12 (0.01 g/kg), vitamin B5 (4.6 g/kg), vitamin C (12 g/kg), folic acid (0.28 g/kg), vitamin 7 (0.4 g/kg), coper (12 g/kg), manganese (40 g/kg), zinc (3.2 g/kg), iron (42 g/kg), iodine (0.8 g/kg), cobalt (0.8 g/kg), selenium (0.35 g/kg)*.

d *Sulfur Cube Was Derived From Commercial Purchase (Sand Sea Sun Shop: TG-6731, Bangkok, Thailand) and Ground (Sieve Size of 1 mm)*.

### Feed sampling and analysis

Every 14 d, samples of dietary ingredients, two experimental diets, and feed refusals were collected, dried at 55°C in an air oven, and pulverized in a Wiley Mill using a (Retsch SM 100 mill; Retsch Gmbh, Haan, Germany) with a 1-mm screen. The dried samples were kept in order to be analyzed for chemical and nutritional content. The nitrogen content of two experimental diets and diet refusals was measured with a KjeltecTM 8400 fully automated Kjeldahl analyser (FOSS, Hilleroed, Denmark); 6.25 was used as the conversion factor to get crude protein (CP) values. The contents of neutral detergent fiber (NDF, with heat-stable α-amylase), acid detergent fiber (ADF), and acid detergent lignin (ADL) were determined using a fully automated system according to Van Soest et al. (19). Hemicellulose content was determined by subtracting NDF from ADF, while cellulose content was determined by subtracting ADF from ADL. Further, the second subsample was extracted at 50°C for 24 h with 0.01 N hydrochloric acid (HCl) dissolved in 80% methanol (14), and the supernatant was transferred to a 50-mL volumetric flask for HPLC analysis of anthocyanin composition. The chromatographic separation was performed in quadruplicates using a reversed-phase Zorbax SB-C18 (3.5 μm particle size, i.d. 4.6 × 250 mm, Agilent Technologies, Santa Clara, CA, USA) for 65 min at 28°C and evaluated using a photodiode array UV detector set to 520 nm (20–22). To note, when the measured nutrient values of the diet were different from the initial values, the formula for the diet was changed to meet the measured nutrient values.

### Blood sampling and analysis

Blood samples were collected 2 h after morning feeding at 09:00 on the last feeding week through jugular venepuncture into a single 10-mL heparin-containing vacuum tube. Plasma concentrations of urea, total protein, glucose, insulin, triglycerides, alanine transaminase, and aspartate aminotransferase were measured in quadruplicate using an automated enzymatic colorimetric approach on a Cobas Integra 400 Instrument (Roche Diagnostics, Mannheim, Germany), as given by Suong et al. (9). Remaining plasma was then utilized to assess plasma antioxidant (TAC, TBARS, SOD, CAT, GSH-Px and GSH-Rx) utilizing an automated enzymatic colorimetric approach on a Microplate (96 wells, UV plate), quadruplicate, equipped into microreader (Varioskan-LUX multimode microplate reader, Thermo Scientific, USA), as described in prior research (8).

### Rumen fluid sampling and analysis

At the end of the experiment, all 40 goats were slaughtered. Immediately, samples (about 500 mL, mixture of liquid and solid) were collected from the dorsal, central, and ventral sections of the rumen to produce a composite sample, which was then strained through four layers of cheesecloth to extract rumen fluid. The pH of the rumen fluid was then immediately measured using a portable pH meter. The strained rumen fluid was then transferred to the laboratory in a sterilizing thermos flask. The filtered rumen fluid was separated into two aliquots upon arrival in the laboratory. The first aliquot of the filtrates, which was 5 mL, was treated with 0.5 mL of HCl with a concentration of 50% (v/v), 0.5 mL of a metaphosphoric acid solution with a concentration of 187.5 g/L, and a solution of formic acid (250 mL), and then it was placed in a freezer at −18°C until the measurements of the chemical analysis of the ammonia-N and volatile fatty acid (VFA). The analyses for ammonia N determined using a micro-Kjeldahl method [Kjeltec 8100, Hillerd, Denmark, (23, 24)] and VFA determined using a gas chromatography (Agilent 6890 GC, Agilent Technologies, Wilmington, DE, USA) with a 30 m × 0.25 mm × 0.25 μm column [DB-FFAP; (25)] were conducted in quadruplicate, and the mean result was used for statistical analysis. The second aliquot of the filtrates (5 mL) was homogenized for microbiological detection and stored at −80°C until the relative abundances of specific rumen bacteria were investigated. Preparation, extraction, and quantification of specific rumen bacteria DNA (including primers) were carried out in accordance with past observation (9). References for the relative abundances of total bacteria, *Ruminococcus albus, Ruminococcus flavefaciens, Fibrobacter succinogenes, Butyrivibrio fibrisolvens, Megasphaera elsdenii, Streptococus bovis*, methanogen, and protozoa were obtained from Vivantis Technologies Sdn Bhd (Selangor Darul Ehsan, Malaysia). To ensure consistency, quantitative real-time PCR assays were done in quadruplicate using both standards and genomic DNA samples for each specified species or group of bacteria. The Ct values were translated into normalized relative numbers using Roche Applied Science's LightCycler 480 software version 1.2.9.11 (Basel, Switzerland), which adjusted for PCR efficiency. The values for the 16S rRNA gene of a given microorganism species or group are expressed as a percentage of total bacteria.

### Carcass and meat measurements

Following animal handling management by Suong et al. (9), goats were fasted for 24 h at the ending of the routine feeding trail before being loaded (at 0500 h) and transported half a mile to a modified university slaughterhouse (F14 equipment building, Suranaree University of Technology, Nakhon Ratchasima, Thailand). Before fasting, the final BW of each goat was determined in order to define the dressing percentage. On the day of slaughter, the hot carcass weight was calculated, and the carcasses were subsequently stored at 4°C for 48 h. The weight of cold carcasses and the weight of shrinking carcasses were determined. Carcasses were sliced up between the 12th and 13th ribs to assess carcass quality characteristics and yield grade indicators. Characteristics of carcass quality included fat thickness at the 12th rib, longissimus muscle (LM) area, intramuscular fat, and final pH values. The carcass color was determined by using a portable Chroma Meter to measure the L^*^, a^*^, and b^*^ color values on the cut lean surface and carcass external fat along the lateral side of the carcass (CR-300, Minolta Corporation, Osaka, Japan). Color measurements were taken in quadruplicate in the CIELAB color spaces L^*^ (0 = black, 100 = white), a^*^ (negative values = green, positive values = red), and b^*^ (negative values = blue, positive values = yellow); large portions of connective tissue and intramuscular fat were removed. The operational handbook (26) was used to determine color saturation.

Meat samples were stripped of external fat and connective tissue to measure ash, protein, moisture, and ether extractable lipid following a previous study (9). Tenderness values of meat samples were expressed as the Warner-Bratzler shear force (WBSF) and determined using the AMSA (1995) recommendations with slight modifications (9). The analyses of meat samples were carried out in quadruplicate, and the mean data was used for statistical analysis.

### Statistical analysis

Animal performance, blood biochemical indices, rumen fermentation, microbial community, carcass characteristics, and meat quality were analyzed statistically using the SAS 9.4 general linear model procedure and the following model: Y_ij_ = μ + τ_i_ + ε_ij_, where Y_ij_ is the response variable, μ is the overall mean, τ_i_ is the experimental diet I = ABS or ABSF), and ij is the residual error. Diet was considered a fixed factor in this model, while the animal was considered a random variable. Using the Shapiro–Wilk test, all data were normalized. The student's *t*-test was used to evaluate all of the data. The least-squares means were reported, and a significance level of *P* < 0.05 was declared for all analyses.

## Results

### Experimental diets, feed intake and growth performance

The chemical composition and fermentation characteristics of anthocyanin-rich black cane silage (ABS) and anthocyanin-rich black cane silage treated with ferrous sulfate heptahydrate (FSH, ABSF) after 90 d of ensiling were distinct (Supplementary Table 1). However, such silages can be used as functional roughage sources in otherwise isocaloric and isonitrogenous experimental diets (Table 1). The hemicellulose and cellulose contents, but not the lignin content, were comparable between the two experimental diets. Furthermore, the anthocyanin content of ABSF was found to be nearly 1.4 times higher than that of ABS. In the anthocyanin fraction, the difference between ABSF and ABS was greatest for the malvidin fraction, which was 1.8 times greater in ABSF than in ABS (Table 1).

Table 2 summarizes the DMI, ADG, and the ratio of ADG to DMI of crossbred Thai-native Anglo-Nubian male goats. After 90 days of routine feeding, the ABSF diet had the same effect on animal growth performance as the ABS it replaced (*P* > 0.05).

**Table 2:** Growth performance of goats fed a total mixed ration supplemented with anthocyanin-rich black cane silage (ABS) or anthocyanin-rich black cane silage treated with ferrous sulfate heptahydrate (ABSF).

**Item^a^**	**Experimental diet**		**SEM**	***P-*value**
	**ABS**	**ABSF**		
Animal number	20	20		
Initial body weight (kg)	14.44	14.40	0.323	0.493
Final body weight (kg)	17.67	18.03	0.503	0.181
ADG (g/d)	35.93	40.33	0.540	0.444
DMI (g/d)	456.61	473.47	0.610	0.175
ADG/DMI	0.08	0.09	0.315	0.625

a *ADG, average daily weight gain; DMI, dry matter intake; SEM, standard error of mean*.

### Blood biochemical indices

The ABSF diet had no effect (*P* > 0.05) on plasma concentrations of glucose, albumin, cholesterol, insulin, triglycerides, HDL, LDL, VLDL, or plasma urea nitrogen (Table 3). Likewise, no significant differences in IgG, alanine transaminase, or aspartate aminotransferase concentrations were found between the two groups (*P* > 0.05). The concentration of TBARS, on the other hand, was ~ 10% lower (*P* < 0.05) when the goats were fed ABSF (Table 3). In contrast, feeding ABSF instead of ABS resulted in higher plasma TAC concentrations (*P* < 0.05) and higher SOD, CAT, GSH-Px, and GSH-Rx activities (*P* < 0.05).

**Table 3:** Blood biochemical indices of goats fed a total mixed ration supplemented with anthocyanin-rich black cane silage (ABS) or anthocyanin-rich black cane silage treated with ferrous sulfate heptahydrate (ABSF).

**Item^a^**	**Experimental diet**		**SEM**	***P-*value**
	**ABS**	**ABSF**		
Total protein (g/L)	72.49	71.09	0.822	0.266
Albumin (g/L)	33.35	32.70	0.659	0.118
Globulin (g/L)	39.14	38.39	0.749	0.190
Blood urea N (mmol/L)	8.01	8.00	0.307	0.778
Insulin (μU/mL)	1.72	1.79	0.490	0.195
Glucose (mmol/L)	5.13	5.35	0.720	0.454
Total cholesterol (mmol/L)	3.12	2.81	0.688	0.075
Triglyceride (mmol/L)	0.57	0.47	0.102	0.287
HDL (mmol/L)	1.72	1.90	0.285	0.129
LDL (mmol/L)	1.02	1.12	0.429	0.420
VLDL (mmol/L)	1.14	0.93	0.449	0.521
Alanine transaminase (U/L)	29.78	29.91	0.740	0.435
Aspartate aminotransferase (U/L)	34.88	35.02	0.615	0.573
IgG (g/L)	11.99	12.04	0.496	0.757
TAC (nmol/μL)	32.66	39.90	0.680	0.014
SOD (U/mL)	83.85	93.04	0.443	0.001
CAT (U/mL)	68.28	75.62	0.752	0.017
GSH-Px (U/mL)	44.03	48.57	0.332	0.010
GSH-Rx (U/mL)	53.24	63.46	0.381	0.016
TBARS (nmol/mL)	40.20	36.08	0.465	0.039

a *HDL, high density lipoprotein; LDL, low density lipoprotein; VLDL, very low-density lipoprotein; IgG, immunoglobin G; TAC, total antioxidant capacity; SOD, superoxide dismutase; CAT, catalase; GSH-Px, glutathione peroxidase; GSH-Rx, glutathione reductase; TBARS, thiobarbituric acid-reactive substances; SEM, standard error of mean*.

### Rumen fermentation and microbial community

The variables of rumen fermentation are summarized in Table 4. Rumen fluid pH values were comparable between the two groups. The rumen ammonia N concentration was found to be ~ 0.9% lower (*P* < 0.05) when the goats were fed ABSF. When ABSF was fed to the goats, on the other hand, the total VFA concentration was ~ 1.5% higher (*P* < 0.05). Feeding ABSF instead of ABS resulted in higher acetate proportions (*P* < 0.05). ABSF feeding had no effect on the propionate and butyrate proportions (*P* > 0.05). Following the administration of ABSF, the ratio of acetate to propionate was found to be ~ 4.6% higher (*P* < 0.05).

**Table 4:** Rumen fermentation of goats fed a total mixed ration supplemented with anthocyanin-rich black cane silage (ABS) or anthocyanin-rich black cane silage treated with ferrous sulfate heptahydrate (ABSF).

**Item^a^**	**Experimental diet**		**SEM**	***P-*value**
	**ABS**	**ABSF**		
pH value	6.83	6.74	0.200	0.194
Ammonia N (mg/dL)	13.39	13.27	0.076	0.043
Total VFAs (mM)	84.53	85.86	0.565	0.021
**Individual VFA (molar % of total VFAs)**				
Acetate	59.29	60.43	0.461	0.033
Propionate	28.34	27.61	0.779	0.178
Butyrate	12.37	11.96	0.597	0.088
Acetate/Propionate	2.09	2.19	0.173	0.023

a *VFAs, volatile fatty acids; SEM, standard error of mean*.

**Figure 2:**
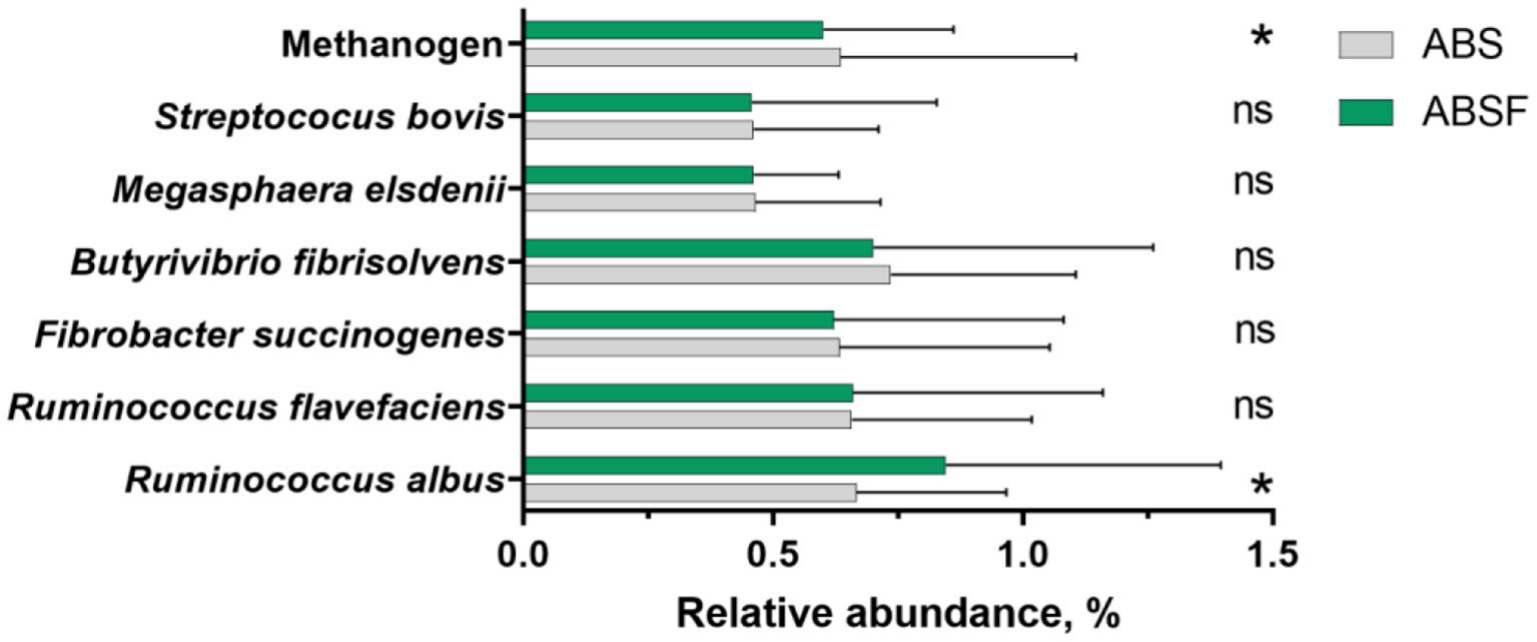
The relative abundances of selected microbial rumen bacteria in goats fed a total mixed ration supplemented with anthocyanin-rich black cane silage (ABS) or anthocyanin-rich black cane silage treated with ferrous sulfate heptahydrate (ABSF). The values are means, with horizontal bars representing standard errors. The values were different (* *P* < 0.05; ^ns^ *P* > 0.05).

The abundances of total bacteria and total protozoa population were not significantly different (*P* > 0.05) between diets. Generally, log_10_ copies of 16 rRNA genes in total protozoa and total bacteria ranged from 6.81–6.94 and 9.47–9.51, respectively. The relative abundances of *R. flavefaciens, F. succinogenes, B. fibrisolvens, M. elsdenii*, and *S. bovis* (Figure 2) were comparable in the two groups (*P* > 0.05). In contrast, when ABSF was fed, the relative abundance of *R. albus* increased (*P* < 0.05), but methanogen decreased (*P* < 0.05).

### Carcass and meat characteristics

The hot and cold carcass weights at slaughter (Table 5) were comparable (*P* > 0.05) between the two diets. Therefore, the dressing percentage was similar between diets (*P* > 0.05). ABSF had no effect on 12th rib fat thickness, LMA, or pH (*P* > 0.05). Also, there were no significant differences in the indices of external fat color or 12th rib lean color between the two groups (*P* > 0.05).

**Table 5:** Carcass characteristics of goats fed a total mixed ration supplemented with anthocyanin-rich black cane silage (ABS) or anthocyanin-rich black cane silage treated with ferrous sulfate heptahydrate (ABSF).

**Item^a^**	**Experimental diet**		**SEM**	***P-*value**
	**ABS**	**ABSF**		
BW (kg)	17.67	18.03	0.503	0.181
HCW (kg)	8.51	8.74	0.796	0.147
CCW (kg)	8.40	8.63	0.963	0.234
DP (%)	48.16	48.47	0.669	0.101
12th–rib fat thickness (cm)	0.233	0.234	0.037	0.407
LMA (cm^2^)	14.52	14.5	0.686	0.556
pH value	6.85	6.83	0.518	0.424
**External fat color**^**b**^, ^**c**^				
L*	22.42	22.49	0.748	0.598
a*	15.09	14.62	0.320	0.279
b*	6.59	6.71	0.420	0.314
c*	16.47	16.08	0.306	0.209
**12th–rib lean color**				
L*	48.74	48.9	0.581	0.321
a*	9.85	9.62	0.401	0.350
b*	6.71	6.88	0.342	0.115
c*	11.92	11.83	0.377	0.097

a *BW, body weight; HCW, hot carcass weight; CCW, cold carcass weight; DP, dressing percentage; LMA, LM area measured at 12^th^ rib; SEM, standard error of mean*.

b *Fat color measurements obtained approximately 20 cm ventrally to the lateral process of the split carcass adjacent the 13^th^ rib*.

c CIE color measurements: L^*^, lightness, black (0) to white (100); positive a^*^, red; negative a^*^, green; positive b^*^, yellow; negative b^*^, blue; c^*^, color saturation = [(a^*^)^2^ + (b^*^)^2^]^1/2^ whereby a large number is considered to be more vivid.

According to Table 6, cooked rate, drip loss, moisture, protein, and ash percentages of steaks were comparable between the two experimental diets (*P* > 0.05). However, after feeding ABSF, the percentage of intramuscular fat in steak samples was found to be ~ 16.8% higher (*P* < 0.05). However, when ABSF was fed, the WBSF value of tenderness was ~ 12.1% lower (*P* < 0.05).

**Table 6:** WBSF and chemical composition of steak samples of goats fed a total mixed ration supplemented with anthocyanin-rich black cane silage (ABS) or anthocyanin-rich black cane silage treated with ferrous sulfate heptahydrate (ABSF).

**Item^a^**	**Experimental diet**		**SEM**	***P-*value**
	**ABS**	**ABSF**		
WBSF (kg)	7.53	6.72	0.468	0.020
Cooked rate (%)	43.02	42.61	0.340	0.282
Drip loss (%)	8.67	11.08	0.490	0.107
Moisture (%)	79.35	78.19	0.351	0.381
Protein (% DM)	88.46	88.27	0.240	0.548
Intramuscular fat (% DM)	5.71	6.86	0.398	0.016
Ash (% DM)	6.18	6.34	0.283	0.083

a *WBSF, Warner-Bratzler shear force values of tenderness; SEM, standard error of mean*.

## Discussion

Anthocyanin is a naturally occurring plant pigment that has beneficial effects not only on the plant itself, but also on other organisms, including humans and animals. In order to accurately provide anthocyanin to ruminant animals via ensiled anthocyanin-rich black cane, it is crucial to investigate the quantitative changes in anthocyanin content during preservation. The majority of the anthocyanin is expected to be lost during silage fermentation. The present findings demonstrated that ensiling anthocyanin-rich black cane without an additive resulted in a loss of anthocyanin, whereas adding 0.030% FSH to anthocyanin-rich black cane prior to ensiling reversed this nutrient loss. It is well-established that FSH can increase the nutritional value of lignocellulosic biomass and reduce anthocyanin loss during roughage preservation for ruminant feed (1, 2). According to the findings of this study, using FSH to preserve roughage appears to be an appealing alternative to developing roughage-based ruminant diets.

The current *in-vivo* results are the first to report on feeding anthocyanin-rich black cane treated with 0.030% FSH (ABSF). The data show that feeding ABSF instead of untreated anthocyanin-black cane (ABS) led to a higher relative abundance of *R. albus*. ABSF, on the other hand, had no effect on the relative abundances of *R. flavefaciens, F. succinogenes, B. fibrisolvens, M. elsdenii*, and *S. bovis*. As a result, ABSF's overall effect on the selected rumen bacteria might be regarded as minimal. This finding appears to be consistent with the findings reported by Tian et al. (12). Indeed, the latter found that supplementary purple corn anthocyanin (PCA) had no effect on the majority of rumen bacteria with a relative abundance of more than 1% at the genus level. Other recent discoveries originated from anthocyanin plant sources, supplementary black cane had no effect on overall bacteria, but anthocyanin content of 0.02% (DM basis) of black cane silage produced an increase in *R. albus*. In the current investigation, the total anthocyanin content of the diet was 0.03% (DM basis), whereas Tian et al. (12) used a maximum PCA content of about 0.14% (DM basis). It is therefore possible to postulate that the total anthocyanin content of the ABSF-diet was insufficient to significantly affect the microbial community. The current findings indicate to verify the prior *in vitro* data published on the FSH-treated black cane-induced improvement of relatively abundance for *R. albus* (2, 9) can be generalized, at least qualitatively, to realistic feeding settings. In addition to this, the feeding of the ABSF led to a decrease in the relative abundance of the methanogens. The observed *R. albus* effects of anthocyanins on lower methanogens are difficult to explain, but the current findings are consistent with findings in sheep and goats that show a reduced relative abundance of methanogens in rumen fluids after feeding mulberry leaf (27) and black cane (9). The drop in the relative abundance of methanogens can be read as methane generation being reduced by feeding ABSF rich in anthocyanin components rise due to residual FSH. Our findings support prior research indicating residual FSH in ABSF appears to impair methanogenesis by making electron exchange more difficult for FSH-reducing bacteria and methanogens harboring Fe oxides (28). This explanation, however, contradicts the reported rise in the rumen acetate to propionate ratio. The latter discovery can be interpreted as ABSF feeding promoting only decreased methane generation *via* reductive acetogenesis (29). Indeed, it is widely accepted that the synthesis of acetate, rather than propionate, provides a source of hydrogen (30, 31). The apparent disparity between the current results on acetic acid and the relative abundance of methanogens is difficult to explain, but it cannot be ruled out that acetogenic bacteria utilized hydrogen to produce acetate. Acetogenic bacteria, in fact, can directly convert carbon dioxide and hydrogen into acetate (32). In this *in-vivo* study, unfortunately, neither the formation of methane nor the quantity of acetogenic bacteria were evaluated, and the results do not provide any further indications to further speculate on the fate of the hydrogen. Future research is thus needed to shed more light on this topic.

Similar to previous studies (2, 9) that observed *in vitro* black cane treated with 0.030% FSH, our findings confirm that those *in vitro* results can be translated to actual feeding situations. Feeding ABSF instead of ABS enhanced overall VFA concentration. The latter result indicates that the ABSF diet made more substrate accessible for fermentation. This could be explained by the role of anthocyanins in bacterial population regulation, as evidenced by the increased relative abundance of *R. albus*. FSH could accelerate the breakdown of the lignin portion in ABSF, increasing the availability of sugar and, as a result, enhancing fermentation acid generation for *R. albus* (2). Despite the fact that we were unable to assess nutritional digestibility during goat digestion, our finding of an increased relative abundance of *R. albus* seemed to support this view. These findings are supported by the findings of Yusuf et al. (33), who revealed that including *Andrographis paniculata* leaves that are high in plant active substances (lactones, anthocyanin, flavonoids, and sterols) in the diet of goats increased the quantity of ruminal *R. albus* while preserving the total bacteria in the ruminal fluid. This inevitably resulted in an improvement in the digestibility of nutrients. According to the current findings, a treated silage-based diet containing 50% ABSF may have a higher rumen bioavailability than an untreated silage-based diet containing 50% ABS. It should be noted that the cellulose, hemicellulose, and CP contents of the two silage-based diets were equivalent, as was the DMI. The idea that ABSF provided more carbohydrates to the ruminal bacteria is supported by the decreased ammonia-N concentrations seen when ABSF was fed. It is generally understood that fermentable carbs play an important role in boosting rumen microbe growth and, as a result, in the conversion of rumen degradable CP to microbial protein (34, 35). Instead, it cannot be ruled out that the increased consumption of anthocyanins led to a reduction in the rumen solubility of the dietary proteins, which in turn led to a reduction in the rumen concentrations of ammonia-N (12, 36). Unfortunately, it is abundantly clear that the current study does not provide any more information on anthocyanin-induced protein solubility reduction, and future research is required to find a solution to this issue. The increased total VFA concentrations induced by the ABSF diet were not related with an increase in DMI or an improvement in the growth performance of meat goats. The significance of the larger overall VFA concentrations can therefore be contested. Unfortunately, neither the fermentable organic matter nor the apparent fecal digestibility of the two diets were measured in the current experiment. As a consequence, any attempt to substantiate the idea that the two experimental diets differ in their ability to be digested by the rumen was hampered. To note, the non-significant effect of dietary ABSF versus dietary ABS on DMI is consistent with previous research (9, 12) on meat goats with anthocyanin content (0.2–1.4 g/head/day). These findings show that an appropriate supplementation of dietary anthocyanin from the experimental diets (ABS or ABSF; ~ 0.3 g/head/day) accounted to ~ 465 g DMI of TMR per day, which is within the usual range for healthy goats.

The current findings support our notion that feeding ABSF reduced the goats' oxidative stress, as seen by lower TBARS values and higher TAC levels. These findings are consistent with prior research as dietary PCA (12), mangosteen peel (37), and piper meal (8). It is possible that the anthocyanins in the ABSF diet, such as cyanidin-3-glucoside, pelargonidin-3-glucoside and malvidin-3-Oglucoside, can explain these findings. These anthocyanins have been shown to fight free radicals and reduce inflammation (9). Simultaneously, feeding ABSF triggered the activity of antioxidant enzymes such as SOD, CAT, and GSH. These results support those of Purba et al. (8), who found modified oxidative markers including SOD, CAT, and GSH activities in ruminal fluid, blood, milk, and mammary tissue in lactating goats fed a total mixed ration comprising piper meal containing flavonoids (potentially including anthocyanin fraction), essential oils, and phenolic acids. Increasing antioxidant enzyme activity in ruminal fluid or blood, on the other hand, may result in a decrease in TBARS levels by optimizing the rumen and its rumen bilayers for dietary bioactive compounds derived from piper meal by boosting membrane fluidity (38). In the current investigation, we hypothesized that increasing the amount of anthocyanin consumed in the ABSF diet could operate as an electron donor, hence mitigating the build-up of reactive oxygen species. Among intracellular enzymes, SOD is the first line of defense against reactive oxygen species scavenging. Activation of CAT and GSH-Px or GSH-Rx, on the other hand, implied that the superoxide anion radical in the dismutation exceeded the limit, resulting in reactive oxygen species being scavenged enzymatically by CAT or GSH before being reduced to water (9). It appeared that SOD, CAT, and GSH were all working together to decrease the scavenging of reactive oxygen species. It would therefore suggest that the lower TBARS values and the higher TAC levels are not only generated by the antioxidative activity of anthocyanin itself, but are also caused by the higher expressions of SOD, CAT, and either GSH-Px or GSH-Rx.

The ABSF feeding had no effect on any of the carcass parameters studied (Table 5, e.g., carcass weight or color), which is similar with recent findings in small ruminants (9, 39), whereas cattle showed a positive effect (40). The disparity in results between research is most likely due to a multitude of factors, including the content of the anthocyanin-based diet utilized in the study, the age of slaughter, and the bioavailability of flavonoids or anthocyanins in ruminants. Additionally, neither the adipose nor the lean tissue color (L^*^, a^*^, b^*^, or c^*^) of goats was modified by the untreated or treated anthocyanin-rich black canes, which indicates that lipid peroxidation was comparable between the two experimental diets. Meat that has less color (a^*^ and b^*^) tends to turn brown, which suggests that the oxymyoglobin to metmyoglobin conversion stage and the interaction with lipid peroxidation were involved in meat turning brown (9, 39). Anthocyanins that contained malvidin and malvidin-3-O-glucoside were able to stabilize muscle membranes, which led to an improvement in the color of the meat (9). Similarly, both the ABS and ABSF diets contain malvidin, cyanidin-3-glucoside, and malvidin-3-O-glucoside, which may prevent lipid peroxidation and retain meat coloring, as we discovered. Moreover, the ABSF diet was associated with increased intramuscular fat contents. The increased intramuscular fat accumulation in goats fed ABSF is most likely due to the increased rumen acetate production. In fact, an increase in acetate production makes more substrate accessible for de *novo* fat synthesis (41). Previous research found a negative relationship between intramuscular fat content and WBSF tenderness values (42). Therefore, it is posited that the lower WBSF values are explained, at least in part, by larger intramuscular fat contents. The ABSF-induced reduced WBSF value is consistent with earlier findings (9, 39).

## Conclusions

The feeding treated anthocyanin-rich black cane with 0.030% ferrous sulfate heptahydrate appears to be an appealing alternative processing method for anthocyanin-rich black cane in meat goat nutrition. The current results show that giving a total mixed ration with 50% treated anthocyanin-rich black cane and 0.030% ferrous sulfate heptahydrate reduces oxidative stress and makes the meat more tender. However, the treated silage-based diet had no effect on dry matter intake or growth performance, and thus could be generalized, at least qualitatively, to realistic feeding settings. In addition, the concentration of total volatile fatty acids and the proportion of acetate were found to be higher in goats fed treated silage-based diets as opposed to untreated silage-based diets, with only a minor effect on the microbial community. The latter could account for both the greater fat content and tenderness of the meat when fed a treated silage-based diet. Research into the impact of the treated silage diet on digestion and carcass traits, particularly the antioxidant profile and the texture of the meat from other animals (cattle, sheep, etc.), is needed to support these findings.

## Data availability statement

The datasets presented in this study can be found in online repositories. The names of the repository/repositories and accession number(s) can be found in the article/Supplementary material.

## Ethics statement

The animal study was reviewed and approved by the Animal Ethics Committee of Suranaree University of Technology issued a statement approving the experimental protocol (SUT 4/2558). The research was carried out in accordance with regulations on animal experimentation and the Guidelines for the Use of Animals in Research as recommended by the National Research Council of Thailand (U1-02632-2559). Written informed consent for participation was not obtained from the owners because the goats are from farm under Suranaree University of Technology and cooperating Thai farmers.

## Author contributions

RP: conceptualization, methodology, formal analysis, investigation, resources, data curation, writing—original draft preparation, writing—review and editing, visualization, supervision, project administration, and funding acquisition. NS: conceptualization, methodology, formal analysis, investigation, resources, data curation, writing—review and editing, visualization, and project administration. SP: conceptualization, methodology, writing—review and editing, supervision, project administration, and funding acquisition. JS: conceptualization, methodology, data curation, and writing—review and editing. PP: conceptualization, methodology, resources, data curation, writing—review and editing, supervision, project administration, and funding acquisition. All authors contributed to the article and approved the submitted version.

## Funding

This work was supported by Suranaree University of Technology (SUT; contract no. Fulltime 61/02/2021), Thailand Science Research and Innovation (TSRI), National Science Research and Innovation Fund (NSRF; project codes: 90464; 160368; FF3-303-65-36-17), National Research Council of Thailand (NRCT; project code: 900105), and Nakhon Ratchasima Rajabhat University (NRRU).

## Conflict of interest

The authors declare that the research was conducted in the absence of any commercial or financial relationships that could be construed as a potential conflict of interest.

## Publisher's note

All claims expressed in this article are solely those of the authors and do not necessarily represent those of their affiliated organizations, or those of the publisher, the editors and the reviewers. Any product that may be evaluated in this article, or claim that may be made by its manufacturer, is not guaranteed or endorsed by the publisher.

## References

[1] Suong NTM Paengkoum S Purba RAP Paengkoum P. Optimizing anthocyanin-rich black cane (Saccharum sinensis Robx) silage for ruminants using molasses and iron sulphate: a sustainable alternative. Fermentation. (2022) 8:248. 10.3390/fermentation8060248

[2] Suong NTM Paengkoum S Salem AZM Paengkoum P Purba RAP. Silage fermentation quality, anthocyanin stability, and in vitro rumen fermentation characteristic of ferrous sulfate heptahydrate-treated black cane (Saccharum sinensis R). Front Vet Sci. (2022) 9:896270. 10.3389/fvets.2022.896270 35656174 PMC9152447

[3] Wang Y Jiang M Zhang Z Sun H. Effects of over-load iron on nutrient digestibility, haemato-biochemistry, rumen fermentation and bacterial communities in sheep. J Anim Physiol Anim Nutr. (2020) 104:32–43. 10.1111/jpn.13225 31663652

[4] Liang JB Paengkoum P. Current status, challenges and the way forward for dairy goat production in Asia - conference summary of dairy goats in Asia. Asian-Australas J Anim Sci. (2019) 32:1233–43. 10.5713/ajas.19.0272 31357264 PMC6668857

[5] Hosseini-Vardanjani SF Rezaei J Karimi-Dehkordi S Rouzbehan Y. Effect of feeding nano-ZnO on performance, rumen fermentation, leukocytes, antioxidant capacity, blood serum enzymes and minerals of ewes. Small Rumin Res. (2020) 191:106170. 10.1016/j.smallrumres.2020.106170

[6] Prentice AM Mendoza YA Pereira D Cerami C Wegmuller R Constable A. Dietary strategies for improving iron status: balancing safety and efficacy. Nutr Rev. (2017) 75:49–60. 10.1093/nutrit/nuw055 27974599 PMC5155616

[7] Salles MSV Zanetti MA Salles FA Titto EAL Conti RMC. Changes in ruminal fermentation and mineral serum level in animals kept in high temperature environments. Rev Bras Zootec. (2010) 39:883–90. 10.1590/S1516-35982010000400025

[8] Purba RAP Paengkoum S Yuangklang C Paengkoum P Salem AZM Liang JB. Mammary gene expressions and oxidative indicators in ruminal fluid, blood, milk, and mammary tissue of dairy goats fed a total mixed ration containing piper meal (Piper betle L). Ital J Anim Sci. (2022) 21:129–41. 10.1080/1828051X.2021.2007173

[9] Suong NTM Paengkoum P Schonewille JT Purba RAP Paengkoum P. Growth performance, blood biochemical indices, rumen bacterial community, and carcass characteristics in goats fed anthocyanin-rich black cane silage. Front Vet Sci. (2022) 9:880838. 10.3389/fvets.2022.880838 35573401 PMC9101464

[10] Wanapat M Chumpawadee S Paengkoum P. Utilization of urea-treated rice straw and whole sugar cane crop as roughage sources for dairy cattle during the dry season. Asian-Australas J Anim Sci. (2000) 13:474–7. 10.5713/ajas.2000.474

[11] Information and communication technology SaPDOoTCaSB. In: Division SaP, editor. Thailand Sugarcane Growing Area Report Production 2004-2020. Bangkok:Office of The Cane and Sugar Board (2021).

[12] Tian XZ Li JX Luo QY Zhou D Long QM Wang X. Effects of purple corn anthocyanin on blood biochemical indexes, ruminal fluid fermentation, and rumen microbiota in goats. Front Vet Sci. (2021) 8:715710. 10.3389/fvets.2021.715710 34589534 PMC8475905

[13] Vlaeminck B Fievez V Cabrita ARJ Fonseca AJM Dewhurst RJ. Factors affecting odd- and branched-chain fatty acids in milk: a review. Anim Feed Sci Tech. (2006) 131:389–417. 10.1016/j.anifeedsci.2006.06.01725228507

[14] Paengkoum S Petlum A Purba RAP Paengkoum P. Protein-binding affinity of various condensed tannin molecular weights from tropical leaf peel. J Appl Pharm Sci. (2021) 11:114–20. 10.7324/JAPS.2021.110314

[15] Mattioli R Francioso A Mosca L Silva P. Anthocyanins: a comprehensive review of their chemical properties and health effects on cardiovascular and neurodegenerative diseases. Molecules. (2020) 25:3809. 10.3390/molecules25173809 32825684 PMC7504512

[16] Suong NTM. Utilization of Anthocyanin-Rich Napier Grass Silage in Growing Goat Diets [Dissertation]. Nakhon Ratchasima: Suranaree University of Technology (2017).

[17] FEEDAP. Safety and efficacy of iron compounds (E1) as feed additives for all animal species: ferrous carbonate; ferric chloride, hexahydrate; ferrous fumarate; ferrous sulphate, heptahydrate; ferrous sulphate, monohydrate; ferrous chelate of amino acids, hydrate; ferrous chelate of glycine, hydrate, based on a dossier submitted by FEFANA asbl. EFSA J. (2016) 14:4396. 10.2903/j.efsa.2016.4396 PMC13093174 42016893

[18] NRC. Nutrition Requirements of Small Ruminants. Washington DC: The National Acadimies Press (2007). p. 8.

[19] Van Soest PJ Robertson JB Lewis BA. Methods for dietary fiber, neutral detergen fiber, and nonstarch polysaccharides in relation to animal nutrition. J Dairy Sci. (1991) 74:3583–97. 10.3168/jds.S0022-0302(91)78551-2 1660498

[20] Purba RAP Paengkoum P. Bioanalytical HPLC method of *Piper betle* L. for quantifying phenolic compound, water-soluble vitamin, and essential oil in five different solvent extracts. J Appl Pharm Sci. (2019) 9:033–9. 10.7324/JAPS.2019.90504

[21] Purba RAP Paengkoum S Paengkoum P. Development of a simple high-performance liquid chromatography-based method to quantify synergistic compounds and their composition in dried leaf extracts of *Piper sarmentosum* Robx. Separations. (2021) 8:152. 10.3390/separations8090152

[22] Tian XZ Lu Q Paengkoum P Paengkoum S. Short communication: Effect of purple corn pigment on change of anthocyanin composition and unsaturated fatty acids during milk storage. J Dairy Sci. (2020) 103:7808–12. 10.3168/jds.2020-18409 32684465

[23] AOAC Horwitz W Latimer GW. Official Methods of Analysis. Gaitherburg, MD: AOAC International Suite 500 (2005).

[24] Paengkoum S Tatsapong P Taethaisong N Sorasak T Purba RAP Paengkoum P. Empirical evaluation and prediction of protein requirements for maintenance and growth of 18–24 months old thai swamp buffaloes. Animals. (2021) 11:1405. 10.3390/ani11051405 34069134 PMC8156132

[25] Purba RAP Yuangklang C Paengkoum S Paengkoum P. Piper oil decreases *in vitro* methane production with shifting ruminal fermentation in a variety of diets. Int J Agric Biol. (2021) 25:231–40. 10.17957/IJAB/15.1661

[26] Minolta C. Precise Color Communication: Color Control From Feeling to Instrumentation. Japan: Minolta (1994).

[27] Ma T Chen DD Tu Y Zhang NF Si BW Diao QY. Dietary supplementation with mulberry leaf flavonoids inhibits methanogenesis in sheep. Anim Sci J. (2017) 88:72–8. 10.1111/asj.12556 27112278

[28] Jin Z Zhao Z Zhang Y. Insight into ferrihydrite effects on methanogenesis in UASB reactors treating high sulfate wastewater: reactor performance and microbial community. Environ Sci Water Res Technol. (2020) 6:1794–803. 10.1039/D0EW00154F

[29] Ungerfeld EM. Metabolic hydrogen flows in rumen fermentation: principles and possibilities of interventions. Front Microbiol. (2020) 11:589. 10.3389/fmicb.2020.00589 32351469 PMC7174568

[30] Purba RAP Yuangklang C Paengkoum P. Enhanced conjugated linoleic acid and biogas production after ruminal fermentation with *Piper betle* L. supplementation. Ciênc Rural. (2020) 50:e20191001. 10.1590/0103-8478cr20191001

[31] Purba RAP Paengkoum S Yuangklang C Paengkoum P. Flavonoids and their aromatic derivatives in *Piper betle* powder promote *in vitro* methane mitigation in a variety of diets. Cienc Agrotec. (2020) 44:e012420. 10.1590/1413-7054202044012420

[32] Katsyv A Müller V. Overcoming energetic barriers in acetogenic C1 conversion. Front Bioeng Biotechnol. (2020) 8:621166. 10.3389/fbioe.2020.621166 33425882 PMC7793690

[33] Yusuf AL Adeyemi KD Samsudin AA Goh YM Alimon AR Sazili AQ. Effects of dietary supplementation of leaves and whole plant of Andrographis paniculata on rumen fermentation, fatty acid composition and microbiota in goats. BMC Vet Res. (2017) 13:349. 10.1186/s12917-017-1223-0 29178910 PMC5701315

[34] Vorlaphim T Paengkoum P Purba RAP Yuangklang C Paengkoum S Schonewille JT. Treatment of rice stubble with *Pleurotus ostreatus* and urea improves the growth performance in slow-growing goats. Animals. (2021) 11:1053. 10.3390/ani11041053 33917899 PMC8068234

[35] Yousefi M Malecky M Zaboli K Najafabadi HJ. *In vitro* and *in sacco* determining the nutritive value of button mushroom stipe and its application in growing lambs diet. Ital J Anim Sci. (2022) 21:279–90. 10.1080/1828051X.2021.1987847

[36] Paengkoum P Liang JB Jelan ZA Basery M. Utilization of steam-treated oil palm fronds in growing saanen goats: II. Supplementation with energy and urea. Asian-Australas J Anim Sci. (2006) 19:1623–31. 10.5713/ajas.2006.1623

[37] Ban C Paengkoum S Yang S Tian XZ Thongpea S Purba RAP. Feeding meat goats mangosteen (Garcinia mangostana L) peel rich in condensed tannins, flavonoids, and cinnamic acid improves growth performance and plasma antioxidant activity under tropical conditions. J Appl Anim Res. (2022) 50:307–15. 10.1080/09712119.2022.2068557

[38] Purba RAP Yuangklang C Paengkoum S Paengkoum P. Milk fatty acid composition, rumen microbial population and animal performance in response to diets rich in linoleic acid supplemented with *Piper betle* leaves in Saanen goats. Anim Prod Sci. (2020). 10.1071/AN20182

[39] Tian XZ Li J Luo Q Wang X Wang T Zhou D. Effects of purple corn anthocyanin on growth performance, meat quality, muscle antioxidant status, and fatty acid profiles in goats. Foods. (2022) 11:1255. 10.3390/foods11091255 35563978 PMC9102689

[40] Prommachart R Cherdthong A Navanukraw C Pongdontri P Taron W Uriyapongson J. Effect of dietary anthocyanin-extracted residue on meat oxidation and fatty acid profile of male dairy cattle. Animals. (2021) 11:322. 10.3390/ani11020322 33525410 PMC7912704

[41] Mu T Hu H Ma Y Feng X Zhang J Gu Y. Regulation of key genes for milk fat synthesis in ruminants. Front nutr. (2021) 8:765147. 10.3389/fnut.2021.765147 34901115 PMC8659261

[42] Arnett EJ Fluharty FL Loerch SC Zerby HN Zinn RA Kuber PS. Effects of forage level in feedlot finishing diets on carcass characteristics and palatability of Jersey beef. J Anim Sci. (2012) 90:960–72. 10.2527/jas.2011-4027 21965452

