# Supplementary material for: Dietary inclusion of anthocyanin-rich black cane silage treated with ferrous sulfate heptahydrate reduces oxidative stress and promotes tender meat production in goats
Source: Front Vet Sci. 2022 Aug 4;9:969321. doi: 10.3389/fvets.2022.969321 (PMC9386371; doi:10.3389/fvets.2022.969321)
Supplement: Supplementary file 1 [file Table_1.DOCX]

Supplementary Material

**TABLE S1 Chemical composition and fermentation characteristic of anthocyanin-rich black cane silage (ABS) and anthocyanin-rich black cane silage treated with ferrous sulphate heptahydrate (ABSF).**

| **Item** | **ABS** | **ABSF** |
| --- | --- | --- |
| Chemical composition (% DM basis, unless otherwise stated) | | |
| Dry matter (% fresh weight basis) | 15.41 | 16.01 |
| Crude protein | 6.42 | 6.48 |
| Neutral detergent fiber | 51.09 | 50.97 |
| Acid detergent fiber | 31.57 | 31.41 |
| Neutral detergent lignin | 3.93 | 2.53 |
| Hemicellulose | 19.52 | 19.56 |
| Cellulose | 27.64 | 28.88 |
| Ash | 11.38 | 12.43 |
| Fermentation characteristic (% DM basis) | | |
| pH value | 4.81 | 4.25 |
| Lactic acid | 3.48 | 4.79 |
| Acetic acid | 2.64 | 2.94 |
| Propionic acid | 0.00 | 0.00 |
| Butyric acid | 0.00 | 0.00 |
| Ammonia nitrogen | 0.03 | 0.03 |
